# Prognostic Impact of Menopausal Hormone Therapy in Breast Cancer Differs According to Tumor Characteristics and Treatment

**DOI:** 10.3389/fonc.2020.00080

**Published:** 2020-02-06

**Authors:** Christopher Godina, Erik Ottander, Helga Tryggvadottir, Signe Borgquist, Karolin Isaksson, Helena Jernström

**Affiliations:** ^1^Department of Clinical Sciences in Lund, Division of Oncology and Pathology, Lund University and Skåne University Hospital, Lund, Sweden; ^2^Department of Oncology, Aarhus University and Aarhus University Hospital, Aarhus, Denmark; ^3^Division of Surgery, Department of Clinical Sciences in Lund, Lund University and Skåne University Hospital, and Central Hospital Kristianstad, Lund, Sweden

**Keywords:** breast cancer, menopausal hormone therapy, prognosis, aromatase inhibitor, cohort

## Abstract

This study investigated how a history of menopausal hormone therapy (MHT) impacts clinical outcomes overall and in different subgroups of breast cancer patients. The study included 814 primary breast cancer patients aged ≥50 years in Sweden (2002–2012) with follow-up until 2016. Associations between patient- and tumor characteristics, recurrences, and overall survival were analyzed in relation to MHT. After a median follow-up of 7 years, 119 recurrences, and 111 deaths occurred. Ever MHT (*n* = 433, 53.2%) was associated with a lower BMI, frequency of alcohol abstinence, and histological grade, higher frequency of oral contraceptive use, and lobular cancer. Overall, MHT was not associated with prognosis, but there were significant effect modifications by estrogen receptor (ER) status, node status, main histological type, and aromatase inhibitor (AI) treatment on recurrence-risk (all *P*_*interactions*_≤ 0.017). MHT conferred an increased recurrence-risk in patients with ER- tumors, adjusted Hazard Ratio (HR_adj_) 3.99 (95% Confidence Interval (CI) 1.40–11.33), in node-negative patients HR_adj_ 1.88 (95% CI 1.11–3.17), and in non-AI-treated patients HR_adj_ 1.81 (95% CI 1.01–3.24), but decreased recurrence-risk in AI-treated patients HR_adj_ 0.46 (95% CI 0.25–0.84) and in patients with lobular cancer HR_adj_ 0.15 (95% CI 0.04–0.64). MHT was associated with lower risk of death in node-positive patients HR_adj_ of 0.48 (95% CI 0.27–0.86) and in AI-treated patients HR_adj_ of 0.41 (95% CI 0.22–0.77), but not in other patients (both *P*_*interactions*_≤ 0.027). A history of MHT may have prognostic value for certain subgroups of breast cancer patients such as AI-treated or node-negative patients.

## Introduction

Breast cancer is the most common cancer among women in Sweden and globally and the second most common cause of cancer death among women in Sweden (1). Menopausal hormone therapy (MHT) is a known risk factor for breast cancer (2–6). Patients are advised to discontinue MHT use at the time of a breast cancer diagnosis. It is still unclear whether there is an association between a history of MHT use and breast cancer prognosis in terms of recurrence rates and overall survival (7), while breast cancer specific mortality appears to be increased (6).

MHT can alleviate menopausal symptoms such as hot flashes and insomnia and was introduced in 1942. During the 1960s MHT became increasingly popular due to the idea that this treatment helped women stay “feminine forever” (8). For many years MHT was widely used and was generally believed to have positive effects on women's health. In 1988, the Federal Drug Administration (FDA) approved the use of MHT to reduce osteoporosis in postmenopausal women (8). However, several years later, clinical trials showed that the health effects of MHT were not as beneficial as was once believed and could even be harmful (9). In 2002, the results of the Women Health Initiative (WHI) randomized controlled trial was published. That study showed that use of combined estrogen and progestin MHT, was associated with an increased risk of coronary heart disease and breast cancer (10). Two large Swedish studies published shortly thereafter showed similar results with respect to the increased risk of breast cancer with use of combined MHT (4, 11). After these results gained media attention, the MHT use markedly decreased (8). Several other studies have reported that MHT increases the risk of breast cancer in women (3, 5, 6, 12–15), specifically when used for more than 4 years (2, 16).

It remains unclear whether a history of MHT has any impact on breast cancer prognosis and if so, to what extent prognosis is affected. Some studies failed to show any significant difference in breast cancer-specific mortality between patients with and without a history of MHT use (5, 17). Other studies have shown an association between previous MHT use and decreased breast cancer mortality (14, 18, 19) and lower risk of death due to any cause (12, 20, 21). In addition, MHT was associated with a reduced risk of breast cancer recurrence in all ages (18) and lower breast cancer specific mortality in patients aged 65 and over (22). As reviewed by Yu et al., MHT prior to breast cancer diagnosis does not appear to be harmful for either overall survival among breast cancer patients or breast cancer specific survival (7, 23, 24). However, the large meta-analysis with over 100 000 postmenopausal breast cancer patients showed an increase in 20-year breast cancer mortality (6). Studies have shown significant differences in patient and tumor characteristics between ever MHT users and those who have never used MHT; however, it is unclear whether differences in prognosis depend on MHT or other factors. MHT has been associated with lower BMI, higher age, smaller tumor size, higher frequency of lobular tumors (22, 25, 26), tumor estrogen receptor (ER) positivity (14, 26), and screening detection (27), i.e., several less aggressive tumor characteristics. In the ATAC study, AI seemed to be associated with somewhat lower recurrence-risk in patients who had used MHT than those who had not (28).

However, to our knowledge there has not been any reports on the prognostic impact of MHT stratified according to type of breast cancer treatment. MHT has in several studies been linked to lobular cancer (25, 26, 29) and one large randomized control trial showed that AI treatment was more effective in patients with lobular cancer (30), but the role of MHT in relation to the prognostic impact of AI-treatment in lobular cancer was not investigated.

Since MHT may be associated with certain patient characteristics and also influence tumor development, we hypothesize that a history of MHT may also influence clinical outcomes differentially according to patient- and tumor characteristics, as well as type of treatment. The aims of this study were to investigate the associations between ever MHT use and patient- and tumor characteristics as well as the prognostic impact in terms of recurrence-risk and overall survival (OS) in different subgroups of breast cancer patients.

## Materials and Methods

**Figure 1:**
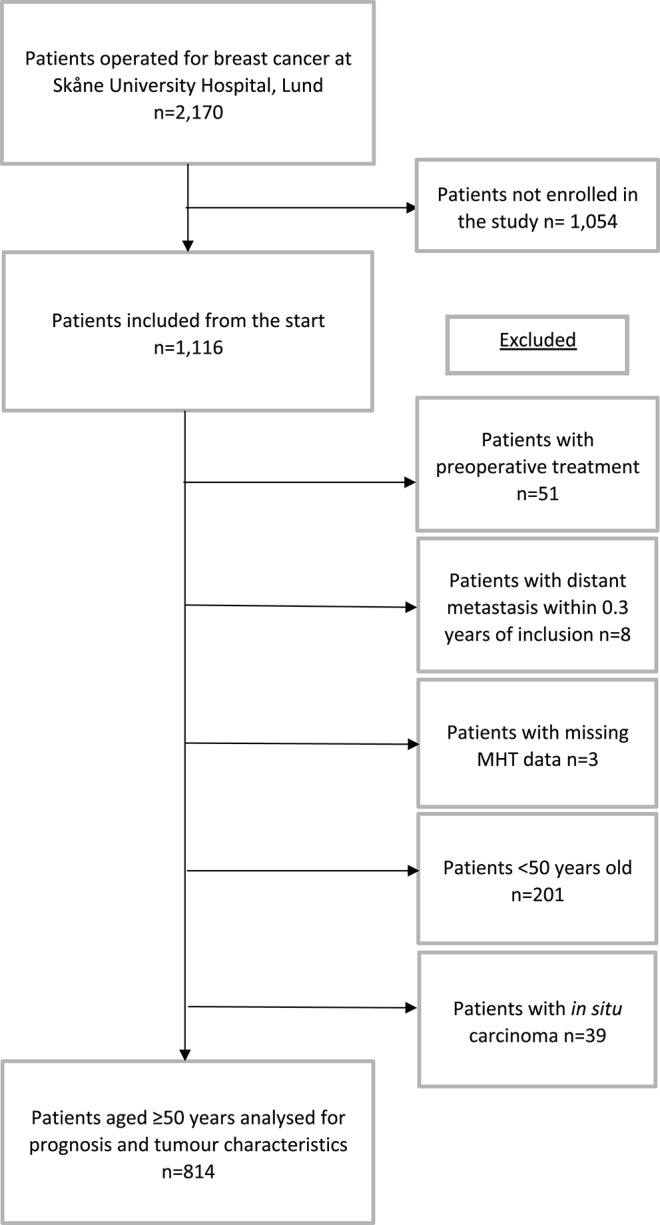
Flowchart of included and excluded patients in this study from October 2002 to June 2012.

The prospective breast cancer cohort BCBlood started in 2002 and is still ongoing. The cohort includes female patients diagnosed with a first breast cancer at Skåne University Hospital in Lund, Sweden. Patients diagnosed with cancer within the 10 years prior to their breast cancer diagnosis were not included in the cohort. The patients described here had all been enrolled in the cohort between October 2002 and June 30, 2012. During this time, 2,170 patients were operated for breast cancer in Lund (31). Of these, 1,116 patients were included in the cohort. After exclusion of patients who had undergone previous preoperative treatment, those who had had an early distant metastasis within 3 months after inclusion, patients with *in situ* carcinoma, patients under age 50 years, and patients who had missing data on MHT use, 814 patients remained (see flowchart in Figure 1). Only patients aged 50 years or older were included because MHT is used to treat menopausal symptoms, and the mean age for reaching menopause is 51 years (32). The Lund university ethics committee approved the study (Dnr LU75-02 with amendments), and all participants signed a written informed consent.

The patients answered a questionnaire preoperatively regarding lifestyle factors such as alcohol intake, coffee intake, smoking habits, reproductive patterns, oral contraceptive use, and MHT use. Several questions were asked regarding MHT use. First, patients were asked whether they had used MHT for menopausal symptoms (yes or no). Second, they were asked whether they were current MHT users (yes or no). Third, the patients reported the duration of their use (<1, 1–2, 3–4, and 5+ years). Finally, they were asked about the type of MHT they used. Approximately 40% of the patients did not remember the specific MHT that they had used, and this variable was therefore not analyzed further.

Patients who reported using progestin containing intrauterine devices were not considered MHT users. Patients who had ever used or were current MHT users were considered ever MHT users regardless of the duration of use. Body measurements including height (cm) and weight (kgs) were measured by the research nurse. Body mass index (BMI) for each patient was calculated as kg/m^2^. Clinical data concerning the tumor characteristics were obtained from the pathology report, and the mode of detection and treatments were obtained from the patient charts and questionnaires. Tumors were considered hormone receptor positive if >10% of the nuclei were stained for ER or progesterone receptor (PR), respectively, as previously described (33). Patients answered follow-up questionnaires post-operatively after 3–6 months, 1, 2, 3 years, and thereafter biannually. Patients were followed for recurrences or death or last follow-up until June 30, 2016. Information on recurrences were obtained from patient charts and deaths from the population registry.

### Statistical Analysis

IBM SPSS statistics version 24 was used for the statistical analyzes. Chi-square test was used for analyzes of differences in patient characteristics between ever MHT users and never MHT users including dichotomized variables: ≥2 cups per day coffee consumption, preoperative smoker, alcohol abstainer, ever use of oral contraceptives, and nulliparity. The non-parametric Mann–Whitney *U*-test was used for analyzes of continuous variables because some of these variables were not normally distributed: age at inclusion, BMI, number of children, and age at first child. Analyzes of tumor characteristics were performed using Chi-square test for all tumor characteristics because they were categorical: ER status, PR status, screening-detected, main histological type (no special type (formerly ductal), lobular or other/mixed), invasive tumor size (pT1, 2, 3, or 4), number of involved axillary lymph nodes (0, 1–3, or 4+), and histological grade (I, II, or III).

Ever MHT use was analyzed in relation to breast cancer recurrences. Breast cancer recurrences included several types such as local or regional recurrence, contralateral breast cancer, or distant metastasis. For breast cancer-free interval (BCFI), the time of follow-up was censored at last follow-up or death prior to July 1, 2016. Univariable analyzes of BCFI and OS were performed using Log-Rank tests and Kaplan–Meier estimates, and the number of patients remaining at each time was obtained using life tables. Crude and adjusted hazard ratios (HR_adj_) with 95% confidence interval (CI) were obtained using Cox proportional hazard regression. Adjustments were made for covariates: age at inclusion (continuous), BMI ≥25 kg/m^2^ (yes), invasive tumor size (≤20 vs. ≥21 mm or muscular or skin involvement), any axillary lymph node involvement (yes), histological grade (III vs. I or II), and ER status (positive). Moreover, MHT use in relation to death due to any cause was also analyzed using the univariable and multivariable models. Subgroup analyzes were stratified by ER status, age ≥65 years, any axillary node involvement, tumor size ≥21 mm or muscular or skin involvement (yes), main histological type (no special type (formerly ductal), lobular, and other or mixed), and histological grade III in the crude and adjusted models. Moreover, the subgroup analyzes of main histological type was further stratified by AI treatment in the patients with ER+ tumors.

Two-way interaction terms between ever MHT use and the following variables were calculated and used in adjusted Cox regression analyzes to investigate potential effect modifications: age (≥65 years), BMI (≥25 kg/m^2^), invasive tumor size (≥21 mm or muscular or skin involvement), any axillary lymph node involvement, histological grade (III), ER+ status, main histological type (no special type (formerly ductal) or lobular or other or mixed), chemotherapy, and radiotherapy. Furthermore, among patients with ER+ tumors, two-way interactions terms between MHT use and aromatase inhibitor (AI) treatment as well as tamoxifen treatment were calculated and used in adjusted Cox regression analyzes.

A power calculation assuming 810 patients accrued over 10 years with 4 years additional follow-up, a median follow-up of 7 years in never users, and a frequency of 50% MHT users showed that the study could detect a true hazard ratio (HR) of 0.78 or 1.31 with 80% power and α of 0.05 (34). All of the statistical tests were two-tailed. *P* < 0.05 were considered statistically significant. Nominal *P*-values without adjustments are presented.

## Results

### Patient Characteristics and History of MHT Use

A total of 814 patients were analyzed in this study. Of the 814 patients, 433 (53.2%) had a history of MHT use or were current users at the time of inclusion while 381 (46.8%) patients had never used MHT prior to inclusion (Figure 1). In the group of ever MHT users, 265 (62.5%) had used MHT for 5 years or longer while 159 (37.5%) reported fewer than 5 years of use. There were significant differences in patient characteristics depending on MHT status. Ever MHT users had a lower BMI and a higher ever use of oral contraceptives vs. never MHT users. Furthermore, ever MHT users were less likely to be alcohol abstainers than never MHT users (see Table 1).

**Table 1:** History of MHT use in relation to patient and tumor characteristics.

	**All patients Median (IQR) or no. of patients (%) *n* = 814 (100%)**	**Missing n**	**Never MHT use Median (IQR) or no. of patients (%) *n* = 381 (46.8%)**	**Ever MHT use Median (IQR) or no. of patients (%) *n* = 433 (53.2%)**	***P*-value**
Ever MHT, yes	433 (53.2%)		0	433 (100%)	
Duration		9			
<1year	47 (5.8%)		0	47 (11.1%)	
1–2 years	48 (6.0%)		0	48 (11.3%)	
3–4 years	64 (8.0%)		0	64 (15.1%)	
5+ years	265 (32.9%)		0	265 (62.5%)	
Age at inclusion, years	63.9 (58.3–69.7)	0	63.0 (56.0–70.4)	64.3 (59.6–69.2)	0.09
BMI, kg/m^2^	25.3 (22.8–28.6)	24	26.0 (23.0–29.4)	24.8 (22.5–27.9)	0.002
Coffee, ≥ 2 cups per day	675 (83.2%)	3	319 (83.9%)	356 (82.6%)	0.61
Pre-diagnostic smoker, yes	158 (19.5%)	2	84 (22.2%)	74 (17.1%)	0.068
Alcohol abstainer, yes	86 (10.6%)	3	49 (12.9%)	37 (8.6%)	0.044
Oral contraceptives, ever	533 (65.6%)	1	222 (58.3%)	311 (72.0%)	<0.001
Nulliparous	96 (11.8%)	1	41 (10.8%)	55 (12.7%)	0.39
Number of children	2 (1–3)	1	2 (1–3)	2 (1–3)	0.35
Age at first child, years	24 (21–28)	101	24 (21–28)	24 (21–27)	0.053
**Receptor status**					
ER+	722 (88.8%)	1	329 (86.6%)	393 (90.8%)	0.059
PR+	569 (70.0%)	1	260 (68.4%)	309 (71.4%)	0.36
**Histological type**		0			0.028
Mainly no special type	652 (80.1%)		316 (82.9%)	336 (77.6%)	
Mainly lobular	104 (12.8%)		36 (9.4%)	68 (15.7%)	
Other/mixed	58 (7.1%)		29 (7.6%)	29 (6.7%)	
**pT (Invasive tumor size)**		0			0.82
1 (≤ 20 mm)	595 (73.1%)		273 (71.7%)	322 (74.4%)	
2 (21–50)	208 (25.6%)		102 (26.8%)	106 (24.5%)	
3 (51+)	9 (1.1%)		5 (1.3%)	4 (0.9%)	
4 (muscular or skin involvement)	2 (0.2%)		1 (0.3%)	1 (0.2%)	
**Axillary node involvement**		2			0.65
0	512 (63.1%)		245 (64.6%)	267 (61.7%)	
1–3	233 (28.7%)		103 (27.2%)	130 (30.0%)	
4+	67 (8.3%)		31 (8.2%)	36 (8.3%)	
**Histological grade**		0			0.014
I	212 (26.0%)		88 (23.1%)	124 (28.6%)	
II	417 (51.2%)		190 (49.9%)	227 (52.4%)	
III	185 (22.7%)		103 (27.0%)	82 (18.9%)	
**Detection mode**					
screening detected	518 (63.6%)	0	249 (65.4%)	269 (62.1%)	0.34

### Tumor Characteristics and History of MHT Use

Lower grade tumors and lobular type breast cancers were more common in the group who had ever used MHT vs. the group who had not. Ever MHT users had a higher tendency to have ER+ tumors compared with never MHT users. Some differences in grade III and ER status along with similar tumor size and nodal status imply that the tumors in ever MHT users were somewhat less aggressive (Table 1).

### Recurrence-Risk According to History of MHT Use

**Figure 2:**
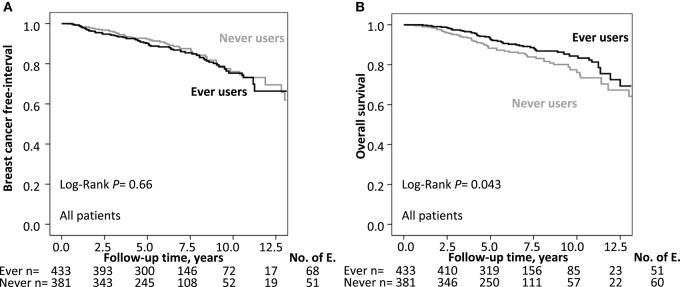
**(A)** Kaplan–Meier estimates of BCFI and ever MHT use. The number of patients is indicated at each follow-up. The study is ongoing, and the number of patients decreases with each follow-up. **(B)** Kaplan Meier estimates of OS and ever MHT use. The number of patients is indicated at each follow-up. The study is ongoing; thus, the number of patients decreases with each follow-up.

The median follow-up time for the 643 patients still at risk was 7.0 years with an interquartile range of 4.9–9.0 years. A total of 119 patients had a breast cancer recurrence: 68 ever MHT users and 51 never MHT users. Kaplan–Meier analyzes of BCFI in relation to history of MHT use showed no significant differences between the groups in terms of OS (Log-Rank *P* = 0.66; Figure 2). The crude HR for a breast cancer recurrence was 1.08 (95% CI 0.75–1.56) while the HR_adj_ was 1.21 (95% CI 0.83–1.77) for ever MHT users. There were significant effect modifications between any MHT use and recurrence-risk depending on ER status, axillary lymph node involvement, main histological type, and AI-treatment; this was not seen with other patient, tumor, or treatment-related factors.

**Figure 3:**
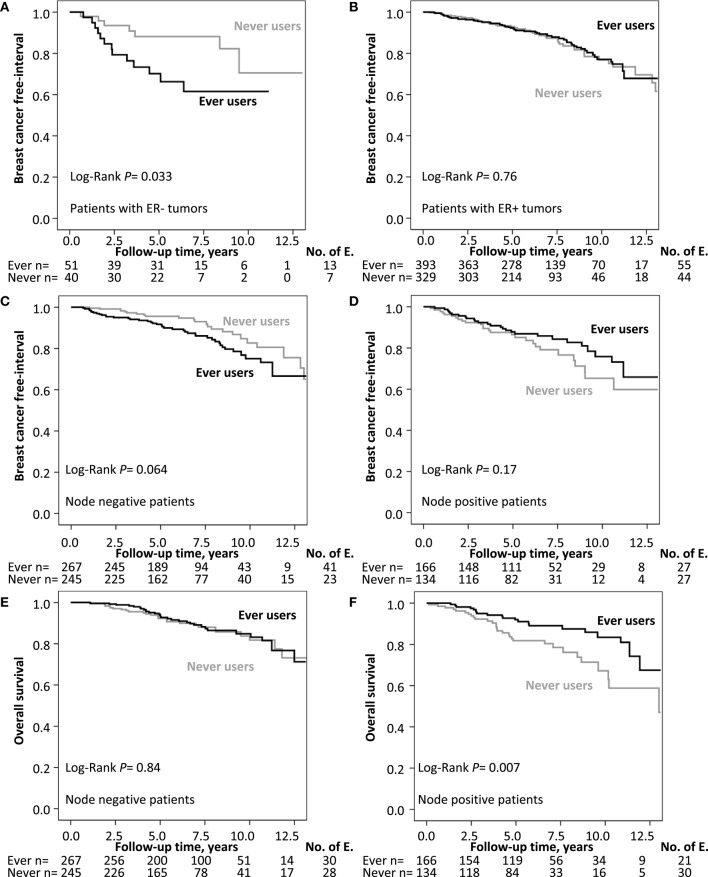
**(A,B)** Kaplan–Meier estimates of BCFI and ever MHT use stratified by ER status. **(C,D)** Kaplan–Meier estimates of BCFI and ever MHT use stratified by axillary node involvement. **(E,F)** Kaplan–Meier estimates of OS and ever MHT use stratified by axillary node involvement. The number of patients is indicated at each follow-up. The study is ongoing; thus, the number of patients decreases with each follow-up.

**Figure 4:**
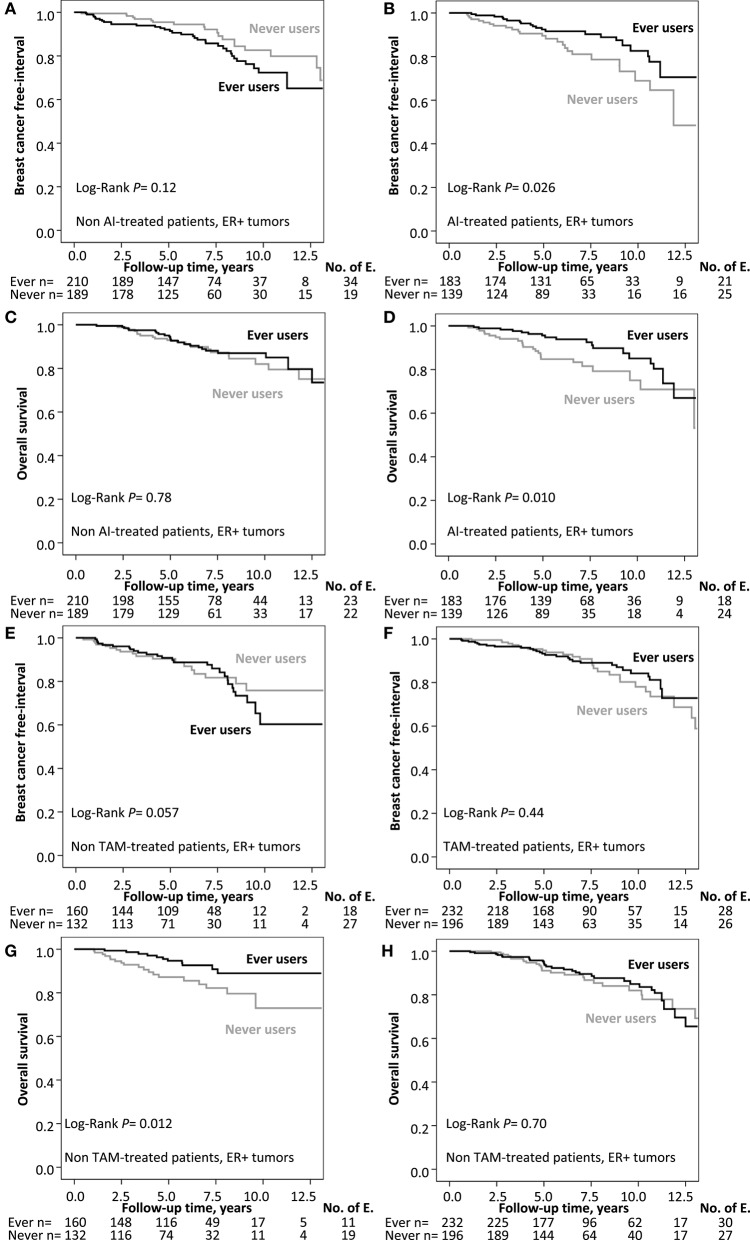
**(A,B)** Kaplan–Meier estimates of BCFI and ever MHT use stratified by ever AI treatment. **(C,D)** Kaplan–Meier estimates of OS and ever MHT use stratified by ever AI treatment. **(E,F)** Kaplan–Meier estimates of BCFI and ever MHT use stratified by ever tamoxifen treatment. **(G,H)** Kaplan–Meier estimates of OS and ever MHT use stratified by ever tamoxifen treatment. The number of patients is indicated at each follow-up. The study is ongoing; thus, the number of patients decreases with each follow-up.

Ever MHT use was associated with an increased recurrence-risk only in patients with ER- tumors, HR_adj_ 3.99 (95% CI 1.40–11.33), but not in patients with ER+ tumors (adjusted *P*_interaction_ = 0.008). Furthermore, ever MHT use was associated with an increased recurrence-risk in node-negative patients, HR_adj_ 1.88 (95% CI 1.11–3.17), but not in node-positive patients HR_adj_ 0.72 (95% CI 0.41–1.27; adjusted *P*_interaction_ = 0.017; Figure 3). Ever MHT use was also associated with lower recurrence-risk in patients with lobular cancer HR_adj_ 0.15 (95% CI 0.04–0.64) but not in tumors with other histological types (adjusted *P*_interaction_ = 0.005). Furthermore, ever MHT use was associated with a lower recurrence-risk in AI-treated patients with ER+ tumors, HR_adj_ 0.46 (95% CI 0.25–0.84) but not in non-AI-treated patients where the risk was increased, HR_adj_ 1.81 (95% CI 1.01–3.24; adjusted *P*_interaction_ = 0.002), see Table 2 and Figure 4. AI-treated patients with lobular cancer who ever used MHT had a lower recurrence-risk than never MHT users HR_adj_ 0.14 (95% CI 0.02–0.82), a similar effect size of ever MHT was seen in non-AI-treated patients with lobular cancer HR_adj_ 0.19 (95% CI 0.01–2.51). In non-AI-treated patients with ER+ tumors and ductal cancer, ever MHT users had an increased recurrence-risk HR_adj_ 2.42 (95% CI 1.24–4.74) but not in AI-treated patients with ductal cancer HR_adj_ 0.56 (95% CI 0.29–1.08). The interaction between MHT and AI was still significant after adjustment for main histological type, however, the association between prognosis and main histological type was not significant after adjustment.

**Table 2:** Multivariable Cox regression analyses of BCFI and OS in (A) all patients and stratified by (B) nodal status, (C) ER status, and (D) AI treatment in patients with ER+ tumors.

**A**					
		**Recurrence-risk HR (95% CI)**		**Death due to any cause HR (95% CI)**	
	Ever MHT, yes	1.21 (0.83–1.77)		0.81 (0.55–1.19)	
	Age, years	1.00 (0.98–1.03)		1.06 (1.03–1.08)	
	BMI, ≥25 kg/m^2^	1.54 (1.06–2.25)		2.00 (1.32–3.04)	
	Invasive tumor size > 20 mm or muscular or skin involvement, yes	1.75 (1.18–2.59)		1.60 (1.07–2.40)	
	Axillary lymph node positive, yes	1.33 (0.91–1.94)		1.54 (1.04–2.29)	
	Histological grade III, yes	1.06 (0.65–1.72)		1.11 (0.68–1.82)	
	ER+, yes	0.52 (0.30–0.92)		0.45 (0.26–0.76)	
**B**					
	**Axillary lymph node status**	**Node positive HR (95% CI)**	**Node negative HR (95% CI)**	**Node positive HR (95% CI)**	**Node negative HR (95% CI)**
	Ever MHT, yes	0.72 (0.41–1.27)	1.88 (1.11–3.17)	0.48 (0.27–0.86)	1.27 (0.74–2.17)
	Age, years	1.02 (0.99–1.06)	0.99 (0.95–1.02)	1.05 (1.02–1.08)	1.07 (1.03–1.11)
	BMI, ≥ 25 kg/m^2^	1.84 (1.01–3.34)	1.36 (0.82–2.24)	1.81 (0.97–3.41)	2.14 (1.22–3.73)
	Invasive tumor size > 20 mm or muscular or skin involvement, yes	1.71 (0.99–2.95)	1.99 (1.11–3.56)	1.57 (0.89–2.76)	1.69 (0.94–3.03)
	Histological grade III, yes	1.22 (0.64–2.35)	0.82 (0.39–1.71)	0.93 (0.45–1.90)	1.29 (0.65–2.54)
	ER+, yes	0.72 (0.32–1.66)	0.38 (0.18–0.83)	0.56 (0.25–1.28)	0.36 (0.18–0.74)
**C**					
	**ER status**	**ER positive HR (95% CI)**	**ER negative HR (95% CI)**	**ER positive HR (95% CI)**	**ER negative HR (95% CI)**
	Ever MHT, yes	0.95 (0.63–1.42)	3.99 (1.40–11.33)	0.72 (0.47–1.12)	1.18 (0.50–2.79)
	Age, years	1.01 (0.98–1.03)	0.98 (0.90–1.05)	1.05 (1.03–1.08)	1.06 (1.00–1.13)
	BMI, ≥ 25 kg/m^2^	1.58 (1.05–2.39)	1.68 (0.63–4.51)	1.99 (1.25–3.17)	2.07 (0.78–5.47)
	Invasive tumor size > 20 mm or muscular or skin involvement, yes	1.53 (0.99–2.37)	3.27 (1.23–8.70)	1.42 (0.89–2.26)	2.24 (0.92–5.46)
	Axillary lymph node positive, yes	1.44 (0.95–2.19)	1.38 (0.55–3.49)	1.76 (1.12–2.76)	1.13 (0.48–2.63)
	Histological grade III, yes	1.25 (0.73–2.12)	0.82 (0.31–2.18)	1.13 (0.62–2.03)	1.30 (0.51–3.32)
**D**					
	**AI treatment, ER+ only HR (95% CI)**	**AI-treated HR (95% CI)**	**Non-AI-treated HR (95% CI)**	**AI-treated HR (95% CI)**	**Non-AI-treated HR (95% CI)**
	Ever MHT, yes	0.46 (0.25–0.84)	1.81 (1.01–3.24)	0.41 (0.22–0.77)	1.23 (0.65–2.30)
	Age, years	1.03 (0.99–1.07)	0.99 (0.95–1.03)	1.06 (1.02–1.10)	1.05 (1.01–1.09)
	BMI, ≥25 kg/m^2^	1.66 (0.88–3.13)	1.72 (0.98–3.02)	2.16 (1.07–4.37)	2.00 (1.05–3.80)
	Invasive tumor size > 20 mm or muscular or skin involvement, yes	1.78 (0.98–3.22)	1.56 (0.78–3.11)	1.65 (0.89–3.07)	1.30 (0.61–2.77)
	Axillary lymph node positive, yes	2.07 (0.98–4.39)	2.23 (1.02–4.86)	1.38 (0.64–2.80)	3.51 (1.63–7.54)
	Histological grade III, yes	1.65 (0.85–3.21)	0.84 (0.29–2.44)	1.48 (0.73–3.01)	0.62 (0.18–2.18)

### Overall Survival According to History of MHT Use

During follow-up, a total of 111 patients died of which 59 patients (47%) had a previous recurrence. There were 51 deaths in ever MHT users and 60 deaths among never users. Kaplan–Meier analysis showed a significant association between ever MHT use and a longer OS vs. patients who never used MHT (Log-Rank *P* = 0.043; Figure 2). The crude HR for ever MHT use was 0.68 (95% CI 0.48–0.99) compared with never use. However, when adjusted for covariates, the statistical analysis showed no significant difference in survival between ever MHT users and never MHT users, HR_adj_ 0.81 (95% CI 0.55–1.19). There were significant effect modifications between ever MHT use and OS depending on axillary lymph node involvement and AI treatment but not with other patient, tumor, or treatment related factors.

Ever MHT use was associated with lower risk of death in node-positive patients with a HR_adj_ of 0.48 (95% CI 0.27–0.86) but not in node-negative patients, HR_adj_ of 1.27 (95% CI 0.74–2.17; adjusted *P*_interaction_ = 0.027; Figure 3). Moreover, any MHT use was associated with a lower risk of death in AI-treated patients with a HR_adj_ of 0.41 (95% CI 0.22–0.77), but not in non-AI-treated patients HR_adj_ of 1.23 (95% CI 0.65–2.30), adjusted *P*_interaction_ = 0.015), see Table 2 and Figure 4.

## Discussion

A history of MHT use seemed to impact clinical outcomes significantly differently according to axillary nodal status, ER status, main histological type, and type of endocrine therapy in breast cancer patients. In the subgroup of AI-treated patients, ever MHT use was associated with significantly better prognosis compared with never MHT use; no difference was seen in tamoxifen-treated patients according to MHT history. For the entire cohort, there was no impact of ever MHT use on prognosis in terms of recurrence or OS when adjusted for covariates. However, MHT use was associated with several patient- and tumor characteristics known to be associated with better clinical outcomes.

The findings regarding patient characteristics are mainly in line with previous epidemiological studies. Ever MHT users had a lower BMI than never users, which has been shown previously (14). The reason for this remains unclear, but it seems to be associated with lifestyle. A previous study showed that there are differences between the groups in lifestyle factors such as total physical activity each week that which could potentially explain the BMI difference (18). In the present study, ever MHT users were significantly less likely to be alcohol abstainers and had a higher tendency to be non-smokers compared to never users, which agrees with a previous study (18). Ever MHT users were also more likely to have used oral contraceptives. We could not find any prior reports on this association but hypothesize that women who already used exogenous hormones in the form of oral contraceptives were likely to use other types of exogenous hormones as well.

There was a significant difference in the frequency of lobular cancer among ever MHT users compared with never MHT users. This is in line with previous research showing that MHT use is associated with a higher frequency of lobular cancer vs. never use (25, 26, 29).

Only a tendency toward a higher frequency of ER+ tumors in ever MHT users was observed, which was quite surprising because earlier studies showed a clear association between MHT use and ER+ tumors (18, 35). Our findings might be due to not making a distinction between current users of MHT at the time of diagnosis and previous users: It has previously been shown that current users have a stronger correlation with ER+ tumors (18). In addition, stratification by the duration of MHT was not performed in the present study. The duration could affect the likelihood of developing an ER+ tumor among MHT users (16).

There was no association between ever MHT use and breast cancer recurrence either in the crude or adjusted models. A previous study also failed to show any significant difference in the recurrence rate between ever MHT users and never MHT users (36). There was no association between MHT use and the overall recurrence rate of breast cancer. However, there was a significant effect modification by ER status. After stratification by ER status, a history of MHT use was associated with a higher recurrence rate only in patients with ER- tumors, which is consistent with Brewster et al. (37); however, the explanation for this association is unclear. Another Swedish study showed that ever MHT use was associated with a longer OS regardless of ER status (12). This contradicts the higher recurrence rate only in patients with ER- tumors seen here. It is worth noting that since there are few cases in the ER—subgroup and thereby low statistical power in the subgroup analysis, this may result in false positive associations (38).

One study reported that MHT withdrawal can alter the protein expression of several important breast cancer biomarkers in tumor cells after a median withdrawal of only 17 days (39). Prasad et al. suggest that MHT withdrawal may act as an aromatase inhibitor with a rapid decrease in estrogen levels that primarily impact ER+ tumor cells (39). Further, patients are advised to stop MHT when breast cancer is diagnosed. Although we did not have data on when MHT was stopped in relation to surgery, we hypothesize that some patients in the ER- subgroup might have been misclassified regarding ER status if the surgery was performed soon after MHT withdrawal or during current use. A few patients continued MHT use against medical advice.

Some patients would possibly have had ER+ tumors and benefitted from endocrine therapy had the interval been longer between the last dose of MHT and the surgery. More research is needed to fully understand whether a history of MHT use is associated with a better or worse prognosis in breast cancer patients with ER- tumors. There was also an effect modification by axillary lymph node status: A higher risk for recurrence and death due to any cause was only seen in node-negative patients with a history of MHT use. Further, when stratified by AI treatment with or without previous tamoxifen, a history of MHT use was associated with a reduced recurrence-risk among AI-treated patients. A previous study showed that ever MHT use is associated with a tendency toward better prognosis with AI treatment compared to tamoxifen treatment (28). In the present study, patients with ER+ tumors of ductal type who did not receive AI treatment and had a history of MHT use had two-fold recurrence-risk compared with never MHT users, but this was not seen in patients with lobular cancer. The biological reason for this is unclear, and further studies are needed. We hypothesize that MHT use in part explains the better prognosis in AI-treated patients with lobular cancer compared with other subtypes reported by Metzger Filho et al. (30). No further associations were found between ever MHT use and recurrence rate in the other subgroups.

Ever MHT users had significantly longer OS in the crude model as seen in previous studies (27, 40). However, there was no significant difference when adjusted for covariates. A probable explanation would be that ever MHT users as a group differ from never MHT users in terms of patients and tumor characteristics as shown in our cohort. These characteristics may influence the prognosis and could account for some of the difference in OS between ever MHT users and never users in the crude model. Nonetheless, there was an association between ever MHT use and longer OS in the subgroup with positive axillary node involvement.

A previous study showed that current use of estrogen plus progestin at the time of breast cancer diagnosis was associated with a lower breast cancer-specific mortality (18). However, ever MHT with estrogen plus progestin was associated with an increase in breast cancer specific mortality in a large meta-analysis (6). Here, breast cancer-specific survival was not investigated; only ~50% of patients that died had a prior recurrence. After stratification by AI-treatment with or without ever tamoxifen treatment, a history of MHT use was associated with longer OS among patients who had ever used AI. This is in line with our results regarding recurrence rate as well as a previous study (28). We hypothesize that the different impact of MHT on AI treatment compared to other treatment groups may in part depend on differences in patient- and tumor characteristics between MHT users and never users. MHT users had lower BMI, which confers lower endogenous estrogen levels (41), making estrogen deprivation with AI easier. MHT also makes tumor cells hyper dependent on estrogen and thus more vulnerable to estrogen deprivation than tumor cells that only relied on endogenous estrogen. Moreover, an earlier study based on the same cohort found that MHT was associated with breast tumor androgen receptor (AR) positivity and AR positivity conferred better prognosis in AI-treated patients (42). Further studies are required to elucidate the relation between MHT use, AR status, and AI treatment.

A prior study using the same cohort has shown that smoking is associated with a higher risk of recurrence and death among AI-treated patients (31). A different study performed within the BCBlood study showed that ever a history of oral contraceptives use led to a lower risk of recurrence among AI-treated patients than non-users of oral contraceptives (43). The interaction between MHT and AI treatment was still significant when adjusted for smoking and ever oral contraceptive use. Furthermore, there was no association between oral contraceptive use and prognosis when adjusted for MHT in this model, but the association between smoking and prognosis of AI-treated patients remained. However, when stratified by tamoxifen treatment with or without previous AI treatment, ever MHT users had a longer OS in the subgroup with no tamoxifen treatment, but most patients had received AI. There was no difference in OS between ever MHT users and never MHT users among patients with tamoxifen treatment. Tamoxifen treatment seems to abrogate difference in OS between ever MHT users and never MHT users: The difference was more pronounced in ever AI-treated patients. The biological mechanism for this difference remains to be elucidated.

One limitation of this study was that only information on total duration was available but not treatment start and cessation. Other studies have shown that current MHT use at time of diagnosis has a stronger association, vs. past use, with better prognosis in breast cancer patients compared to never users (18, 44). More studies are therefore required on this topic to determine if the time when a patient has used MHT in their life makes any difference on the association between the MHT use and later breast cancer prognosis.

Another limitation was that 40% of MHT users could not recall which type of MHT they used, and the MHT type was therefore not analyzed. Previously, estrogen plus progestin MHT—but not estrogen-only MHT—has been associated with longer survival among breast cancer patients (40). Future studies could therefore be valuable to investigate whether there is a differential prognostic impact in certain subgroups of breast cancer patients depending on the type of MHT. Patients were asked about their MHT use only once—in the questionnaire that they filled out during their first visit. A possible way to ensure the validity regarding type of MHT would have been through the pharmaceutical registry that was established in 2005. However, the first patients were included in 2002, and the registry thus lacked data on MHT use on a large percentage of patients.

Furthermore, it is possible that some patients who did not recall the type of MHT had only taken complementary alternative medicine such as black cohosh containing herbal drugs and reported it as MHT. The black cohosh containing drug “Remifemin” could be prescribed as MHT as of June 30th, 2012 (45). Therefore, this study did not include patients enrolled after June 2012 to minimize the risk for misclassification. Patients that reported that they had only used complementary alternative medicine or a progestin-containing intrauterine device were not classified as ever MHT users.

A previous study using the same cohort as this study showed that the patients included are similar to all breast cancer patients operated at Lund University Hospital during the same period (31). The patients who were not included in the cohort were mainly missed due to a lack of research nurses. The reasons for excluding patients were that preoperative treatment changes tumor characteristics while early metastasizing tumors or *in situ* tumors could lead to misleading results when evaluating prognosis. Therefore, the patients in the study are considered representative of female breast cancer patients operated in Lund. This is a strength of the study. The catchment area for the hospital in Lund includes everyone living in the university town of Lund and also several neighboring municipalities for a total of approximately 300,000 people. The health care conducted at the Lund University Hospital is part of the Swedish health care system, and there are no private clinics performing breast cancer surgeries. However, patients living in other areas might have different lifestyle patterns, and it is uncertain if the results are generalizable to breast cancer patients in Sweden.

The questionnaires were filled out preoperatively when the patients had just received their diagnosis. Therefore, there was no risk of a survivor bias that might be present in studies that recruited patients where the diagnosis occurred several years ago: Only patients that had survived up until that point could answer; other patients would be missed. Furthermore, the study design also minimizes the risk of recall bias between patients who recur and are still in remission because information regarding MHT use was obtained preoperatively. The information about alcohol and smoking habits might have a risk of a bias because people might have understated their alcohol consumption and smoking frequency. However, information regarding factors such as BMI, tumor characteristics, recurrence, and death are reliable. Body measurements were obtained by research nurses and information on tumor characteristics was obtained from the pathology reports. Information about recurrences was obtained from patient charts, and cases of deaths were identified in the Swedish population registry; thus, the database should be nearly complete.

## Conclusion

Overall, there was no difference in recurrence-risk or death between ever MHT users and patients who never used MHT. The difference in OS in the crude model might be explained by differences in patient and tumor characteristics. A history of MHT use was associated with a worse prognosis among node-negative patients and patients with ER- tumors. The mechanisms of these effect modifications are unknown. In addition, a history of MHT use was associated with a significantly better clinical outcome only among AI-treated patients; no difference in clinical outcome according to history of MHT use was observed in tamoxifen-treated patients. Furthermore, a history of MHT use might confer improved prognosis in patients with lobular breast cancer regardless of AI treatment. If these results are confirmed, then they suggest that MHT could have prognostic value for certain subgroups of patients such as AI-treated or node-negative patients.

## Data Availability Statement

The datasets generated for this study will not be made publicly available due to privacy laws.

## Ethics Statement

The studies involving human participants were reviewed and approved by Lund university ethics comittée. The patients/participants provided their written informed consent to participate in this study.

## Author Contributions

CG, EO, and HJ: study design, statistical analysis, data interpretation, data analysis, and interpretation. HT and HJ: data collection. CG, EO, HT, SB, KI, and HJ: manuscript preparation. In addition, all authors confirm they contributed to manuscript reviews—revising it critically for important intellectual content—and read and approved the final draft for submission. All authors are also responsible for the manuscript content.

### Conflict of Interest

HJ holds stocks in Pfizer, SB received honoraria from Pfizer and Roche. The remaining authors declare that the research was conducted in the absence of any commercial or financial relationships that could be construed as a potential conflict of interest.

## Abbreviations

AI: Aromatase Inhibitor
AR, Androgen Receptor, BCFI: Breast cancer free-interval
BMI: Body Mass Index
CI: Confidence interval
ER: Estrogen receptor
FDA: Federal Drug Administration
HR: Hazard ratio
HR_adj_: Adjusted hazard ratio
kg: kilogram
OS: Overall survival
m: meter
MHT: Menopausal hormone therapy
PR: Progesterone receptor
pT: Invasive tumor size
pN: Axillary lymph node involvement
NST: No special type
WHI: Women's Health Initiative
